# Internet of Unmanned Aerial Vehicles: QoS Provisioning in Aerial Ad-Hoc Networks

**DOI:** 10.3390/s20113160

**Published:** 2020-06-02

**Authors:** Kirshna Kumar, Sushil Kumar, Omprakash Kaiwartya, Ajay Sikandar, Rupak Kharel, Jaime Lloret Mauri

**Affiliations:** 1 Jawaharlal Nehru University (JNU), New Delhi 110067, India; kirshn44_scs@jnu.ac.in (K.K.); skdohare@mail.jnu.ac.in (S.K.); 2 School of Science and Technology, Nottingham Trent University, Clifton Campus, Nottingham NG11 8NS, UK; 3 GL Bajaj Institute of Technology & Management, Greater Noida 201306, India; ajay.sikender@glbitm.org; 4 Department of Computing and Mathematics, Manchester Metropolitan University, Manchester M15 6BH, UK; r.kharel@mmu.ac.uk; 5 Integrated Management Coastal Research Institute, Universitat Politecnica de Valencia, 46022 Valencia, Spain; jlloret@dcom.upv.es

**Keywords:** aerial communication, internet of unmanned aerial vehicles, quality of service provisioning, vehicular communications

## Abstract

Aerial ad-hoc networks have the potential to enable smart services while maintaining communication between the ground system and unmanned aerial vehicles (UAV). Previous research has focused on enabling aerial data-centric smart services while integrating the benefits of aerial objects such as UAVs in hostile and non-hostile environments. Quality of service (QoS) provisioning in UAV-assisted communication is a challenging research theme in aerial ad-hoc networks environments. Literature on aerial ad hoc networks lacks cooperative service-oriented modeling for distributed network environments, relying on costly static base station-oriented centralized network environments. Towards this end, this paper proposes a quality of service provisioning framework for a UAV-assisted aerial ad hoc network environment (QSPU) focusing on reliable aerial communication. The UAV’s aerial mobility and service parameters are modelled considering highly dynamic aerial ad-hoc environments. UAV-centric mobility models are utilized to develop a complete aerial routing framework. A comparative performance evaluation demonstrates the benefits of the proposed aerial communication framework. It is evident that QSPU outperforms the state-of-the-art techniques in terms of a number of service-oriented performance metrics in a UAV-assisted aerial ad-hoc network environment.

## 1. Introduction

Unmanned aerial vehicle (UAV)-assisted Internet of Things (IoT) is a growing research theme in smart city-oriented applications [1,2]. The smart service-centric domain includes aerial delivery [3], smart healthcare, smart homes, Internet of vehicles [4], UAV simulation [5], pollution monitoring, smart agriculture, disaster management [6], industrial Internet of Things, and green mobility [7]. It has emerged as a prominent development area to revolutionize UAV-assisted IoT in daily day life [8]. In sensor-enabled UAV-assisted applications, for transmitting and viewing data immediately, the Internet is utilized to connect actuators and sensors inside the aerial vehicles. After the aerial trip completion, flying-related data is tracked in real-time with the usage of IoT devices and technologies for downloading data from sensors [9]. Currently, the usage of UAVs with intelligent sensors is most prominent in agricultural monitoring, such as disease and pest detection of crops. Characteristics such as image definition and high flexibility in sensor-enabled UAVs promote the applications of UAV-assisted IoT to make modern agriculture more intelligent [10,11].

**Figure 1:**
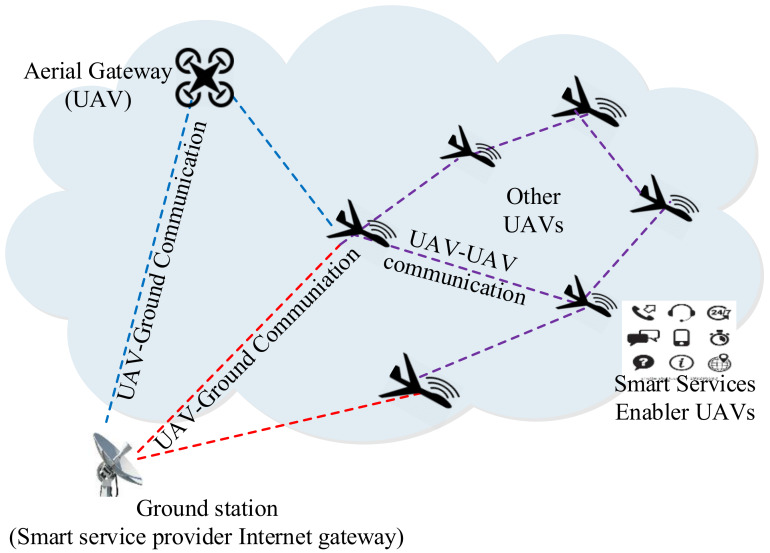
A communication system for unmanned aerial vehicle (UAV)-enabled aerial ad hoc networks (UAANETs).

Figure 1 illustrates the UAV-centric aerial communication environment, including aerial base station-enabled, and ground base station-enabled communications. It is clarified that the aerial-based station is also a UAV, but it is assumed that it is equipped with higher computing and communication capacity than the other UAVs. For differentiation, different types of component figures are used. Further, it is also clarified that most of the computing activities are performed at the ground server and the UAVs are services enablers in such networks. The icons are used to show that smart services are provided by the UAV-enabled aerial ad hoc networks. In an aerial base station-enabled system, sensor-enabled UAVs access the Internet and remain in contact with ground systems via utilizing the base station as a relay node to provide larger coverage in remote, ocean, or polar areas for enabling smart services [12]. However, the aerial base station-based centralized UAV system has higher operation and maintenance costs with lower reliability for enabling smart services due to the dependency on the base station. In a multi-hop ad hoc networking environment of UAVs, extension of the network architecture is known as UAV-enabled aerial ad hoc networks (UAANETs) [13,14]. While maintaining communication among UAVs and ground and aerial base stations over the region, UAANETs can be utilized as a smart service provider infrastructure in smart cities. UAANETs can be deployed to provide Internet accessibility via sensor-enabled UAVs in identified coverage areas without any fixed infrastructure support [15]. Therefore, quality of service (QoS) improvement in UAANETs is a potential step towards an Internet of UAVs for enabling various smart services in both hostile and non-hostile environments. However, providing stable and reliable communication framework for UAANET is a challenging task according to the literature [16].

In this context, this paper proposes a quality of service provisioning framework for a UAV-assisted aerial ad hoc network (QSPU) focusing on reliable aerial communication. The aerial mobility and service parameters of sensor-enabled UAVs are modelled considering highly dynamic aerial ad hoc environments. The parameters include connectivity among UAVs, service delay, lifetime of the connected route, and service overhead in aerial ad hoc networks environments. The key contributions of the paper can be summarized as follows:

- Firstly, an aerial ad hoc mobility model is presented for a UAV-assisted communication framework.
- Secondly, quality of service parameters for UAV-assisted networking are derived focusing on connectivity among UAVs, route lifetime, and delay in service delivery.
- Thirdly, a complete communication framework for quality of service provisioning in UAV-assisted aerial ad hoc networking (QSPU) is developed based on the aerial ad hoc mobility model and service parameters.
- Finally, the performance of the proposed aerial networking framework is evaluated in comparison with the state-of-the-art techniques, considering a number of metrics for aerial ad hoc networking environments.

The remainder of this paper is structured as follows. Section 2 explores the related literature on service provisioning protocols for aerial ad hoc networking environments. Section 3 presents the details of the proposed service provisioning framework, QSPU, for UAV-assisted aerial ad hoc network environments. Section 4 discusses the implementation environments and analysis of results. The conclusion is presented in Section 5.

## 2. Related Work

Mobile networking in the sky was initially introduced considering commercial UAVs as a router [17]. This enables in-flight Internet by addressing two major issues, namely, resource management and Internet over satellite support. A data access architecture, clustering of aerial nodes focusing on stability, and a reliable data dissemination scheme were the major developments of the work. However, it is a novel application of an ad hoc networking scenario in aerial environments. To highlight the significance of ad hoc networks in civilian applications, a Greedy Perimeter Stateless Routing (GPSR) was suggested [18]. GPSR utilizes the one-hop greedy forwarding approach for making the next-hop decision. In the case of unavailability of greedy forwarding nodes, the perimeter of the one-hop transmission region is considered as the forwarding region for data dissemination. This approach was a significant step towards information dissemination in environments without infrastructure and, therefore, a good candidate for aerial ad hoc network environments. Further, the ad hoc on demand vector protocol (AODV) was suggested while selecting the routes on demand on the basis of minimal hop count [19]. In AODV, HELLO (hello) packets are utilized periodically to check the active neighboring nodes. Due to reactive routes, this protocol has a number of advantages, particularly, less overhead and complexity. However, higher delay with higher rate of failure in data delivery are the main concerns because of single route failure. AODV also suffers from the problem of energy holes because of unbalanced energy consumption and unreliable data transmission.

An ad hoc routing scheme has been proposed while exploiting the geo-localization information and proactive functions for the complete and shortest route selection in aerial mobile ad hoc networks [20]. However, this scheme results in less route stability due to consideration of only the hop count or distance as the route selection parameter. A geo-location-based aerial routing technique for highly varying telemetry environments has been suggested, while utilizing node location and wireless channel broadcasting to meet the need of airborne telemetry applications [21]. However, this technique experiences higher end-to-end delay and network congestion. A geographical routing technique has been proposed for UAANETs that eliminates the beaconing of traditional routing with the usage of position and velocity of the UAV [22]. In this technique, the parameter based on velocity is utilized to select the neighboring hop. The performance of this protocol weakens in a network with heavy data traffic. Two novel stability-driven clustering schemes have been proposed for establishing stable clusters for highly mobile ad hoc networks comprising ships, UAVs, cars and trains as mobile nodes [23].

For scenarios with unknown position information of mobile nodes, the first scheme is utilized, and for scenarios with known position information (via GPS), the second scheme is utilized. This scheme lacks data reliability. Geographical routing based on an automatic dependent surveillance broadcast system has been suggested while utilizing UAV position and velocity to remove beaconing of traditional routing [24]. In this scheme, the next hop is selected based on the UAV velocity metric while adaptively coping with highly dynamic UAV and network topology. This scheme does not focus on providing optimized load capacity. Reactive greedy reactive routing has been proposed for unmanned UAANETs with high mobility and variable density while combining the characteristics of reactive routing techniques with geographical routing techniques [25]. In this scheme, a velocity vector-based mobility prediction technique is utilized to predict the UAV location and two various scoped flooding techniques are used to reduce overhead messaging. A UAV-based communication system providing connectivity and deployment modules for emergency disaster recovery has also been proposed [26]. This system comprises three prominent subsystems for navigation, communication, and schemes for formation management. Parameters such as link availability, jitter, throughput, and packet loss were not considered in the communication system.

Link availability estimation-based routing has been proposed while utilizing the link availability parameter for the selection and updating of the route [27]. First, a semi- Markov mobility model was presented to emulate the behavior of airliners, then, the link availability period, pdf of the relative speed between two UAVs, and the expected link lifetime were used to select the reliable route. In this scheme, the relative speed of the derivation of the link availability metric and the pdf of the link lifetime were utilized to select a reliable route. However, the metric of the load balance was not considered. Geographic load share routing (GLSR) has been suggested while utilizing the information related to the UAV and the buffer capacity for exploiting air–ground capability. In this model, a new technique based on Doppler shift was utilized to predict link stability while controlling the data packets. Unfortunately, GLSR only operates with a static topology. However, parameters such as QoS and load balancing are not considered. On the basis of Doppler shift-based GLSR, QoS-based multipath doppler routing (QoSMUDOR) [28] has been suggested, providing enhanced link stability with the usage of QoS metrics. However, in this scheme, only theoretical assessment has been conducted, and aspects related to specific QoS parameters have not been presented.

A delay aware reactive routing technique has been proposed that utilizes an expected node delay parameter for finding an optimized route [29]. Performance parameters of traffic demand and network stability were utilized for the analysis of the scheme. However, load balancing is not considered. A joint internet gateway allocation, scheduling, and routing scheme has been suggested to minimize the average packet delay in mobile aerial ad hoc networks [30]. A mathematical programming scheme was proposed comprising two steps: weighted hop count minimization for scheduling and average delay reduction for routing. Further, a genetic algorithm was been formulated to reduce computational complexity in large mobile networks. However, but this scheme does not provide an optimized link lifetime. A network coding based multi path routing protocol has been suggested to minimize bit error rate and propagation delay inside a space information network [31]. The number of packets received currently and the status of the next hop link are utilized to evaluate the code number of packets that balances the network load. However, this protocol does not optimize link lifetime. A two-dimensional UAANET framework has been proposed for a single flight route while deriving connectivity for various transmission ranges [32].

A hybrid computing model with the integration of edge-cloud computing and a UAV-swarm has been suggested for enhancing the quality of service inside UAV networks [33]. Furthermore, quality of service has been improved by utilizing joint task placement and routing based on a Markov approximation scheme for a latency-critical UAV swarm. This scheme is claimed to reduce latency and operating cost. However, connectivity-centric quality of service monitoring is lacking for overcoming networking dynamism in aerial environments. Am approach based on software-defined networking has been suggested to address the problem of UAVs as data providers considering a highly mobile network for a military surveillance system [34]. This approach is claimed to improve end-user quality of experience. An optimization algorithm based on particle swarm optimization has been explored to reduce total power consumed by base stations and optimizing quality of service in a UAV-based wireless communication system [35]. This algorithm is claimed to reduce deployment and operational costs, but does not consider quality of service in an aerial environment. A framework for multi-agent intercommunication and end-to-end communication has been investigated for UAV-based applications [36]. This framework considered channel approximation based on least squares estimation and uses environmental effects and a gradient-based optimal relay position method. However, the approach does not optimize the quality of service metrics in an aerial environment. A flying ad hoc network enabled by a hybrid communication system for UAVs (HCU), focusing on data rate and power consumption, has been suggested, [37]. An experimental case study has been examined considering two types of wireless communication systems, namely, Wi-Fi and Bluetooth. Wi-Fi was utilized for higher data rate-oriented services and Bluetooth was utilized for low power consumption-oriented services. However, guaranteed quality of service was not considered in the system, reducing the practical application of the hybrid communication system for flying ad hoc networks.

## 3. Quality of Service Provisioning in UAV Assisted Networks

In this section, the proposed quality of service provisioning framework for UAV-assisted networks (QSPU) is presented in detail. In particular, a network model comprising a mobility model for a UAV-assisted ad hoc networking is presented. The service parameters, including UAV connectivity, route-oriented service lifetime, and service delay, are derived. For smart service delivery, a route selection approach based on the service parameters and a broadcasting optimization technique have been developed.

### 3.1. UAV Network Mobility Model

The network model consists of three components: sensor-enabled UAVs, static base stations at the ground, and aerial base stations. Here, ground stations work as IG (Internet gateways). The scenario concerns only the communication between the sensor-enabled UAVs and IGs. For simplification in network modeling, the following assumptions were made:

- It is an ad hoc networking model with a number of independent networks.
- The distribution of all UAVs is done continuously where each UAV joins the network independently.
- The transmission power and physical layer parameters related to all UAVs are uniform.
- An automatic dependent surveillance-broadcast system is considered for all UAVs to acquire real-time state vector such as position, velocity, ID (Identification), and other information.

On the basis of the UAV’s mobility trace in the sky, we can categorize an UAV node movement in to five phases: acceleration phase, steady climb, middle smooth, steady down, and deceleration. Here, it is clarified that mobility trace means the range of location data logged by each UAV when it joins the UAANET. The mobility-oriented set of location data can be categorized to further model the network and provide greater clarity in managing the UAVs presence in the network. In the acceleration phase, the velocity of the UAV increases until it reaches the target velocity $v^{\propto }$. The UAV selects the targeted horizontal direction $\emptyset^{\propto }$ in the range [0, 2π]. In the steady climb phase, the UAV climbs in the target vertical direction $\emptyset^{\propto }$ in the range $\left[0,\;\pi /2\right]$ and moves with constant velocity $v^{\propto }$. Here, $v^{\propto }$ is randomly selected from the range of 650–1000 m/h, which is considered the standard velocity in various UAV models. During the middle smooth phase, the movement of the UAV is steady and smooth according to the Gauss Markov model [38]. Further, in the steady down phase, the velocity of the UAV is equal to $v^{\propto }$. The UAV selects a horizontal direction equal to $\emptyset^{\propto }$ and a vertical direction in the range [π/2, π]. Finally, in deceleration phase, the UAV uniformly decreases its velocity in one direction until it stops. In the initial acceleration phase, the UAV is assumed to take 5 min to join the UAANET. It is also assumed to take 5 min in the final deceleration phase. Here, it is clarified that in the modelling and experiment, small UAVs were considered. This is due to the superior mobility flexibility within the location coordinate range for each UAV, and single smart service support consideration by each UAV. Further, it is also noteworthy that the join delay of 5 min was assumed to allow the clear performance differentiation of the growing number of UAVs in the network. However, the delay might be a longer consideration for small UAVs.

### 3.2. Route Connectivity

Route connectivity of an UAANET is described as the transmission range, when connectivity probability $P^{c}$ is 1 for any ρ (see Table 1).

In this network model, the network of UAVs at the same height level is denoted as a sub-UAANET. Here, a crosslink exists between two UAVs, if these UAVs belong to different sub-UAANETs and are able to communicate. Therefore, we can assume that a transmission range of $T^{1}$ guarantees that the probability of connectivity is almost 1 inside each sub-UAANET. A transmission range of $T^{2}$ guarantees that the probability of connectivity is almost 1 when there exists at least one crosslink between two neighboring sub-UAANETs. The probability of connectivity $P^{c}$ within a sub-UAANET can be computed as:

(1) $$P^{c}={\left[\frac{1-\left(1+\sigma T\right)e^{-\sigma T}}{1-e^{-\sigma T}}\right]}^{\left\lfloor X/T\right\rfloor }\times \left\{1-\left[1+\sigma T\left(X/T-\lfloor X/T\rfloor \right)\right]\right\}e^{-\sigma T}$$

Let

(2) $$T^{1}=\frac{\left(1+\rho \right)\ln\left(\sigma X\right)}{\sigma }$$

where ρ is a small positive parameter. $P^{c}$ can then be expressed as:

(3) $$P^{c}\geq {\left[\frac{1-\left(1+\sigma T\right)e^{-\sigma T}}{1-e^{-\sigma T}}\right]}^{\left\lfloor X/T\right\rfloor }\times \left(1-e^{-\sigma T}\right)=[{\left(1-\beta {)}^{1/\beta }\right]}^{\frac{e^{X}}{{\left(e^{X}\right)}^{1+\rho }-1}\times \left[1-\frac{1}{{\left(e^{X}\right)}^{1+\rho }}\right]}$$

where $\beta =\frac{\sigma T}{e^{\sigma T}-1}$.

We then need to verify that:

(4) $$\underset{T\to \infty }{\lim}P^{c}\geq 1$$

Hence, for sufficient range $T^{1}$, the probability of connectivity is almost 1 inside each sub-UAANET.

**Figure 2:**
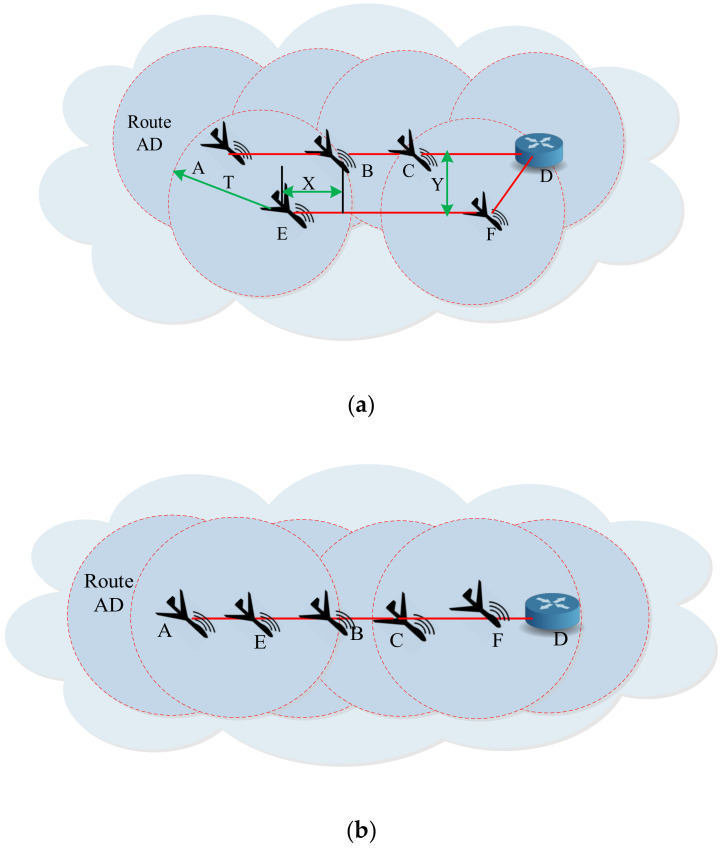
Superposition of neighboring sub UAANETs. Caption of (**a**) communication among UAVs with two levels and (**b**) communications among UAVs with single level.

Assuming there are n crosslinks between two neighboring sub-UAANETs, then according to Figure 2a, if horizontal and vertical distances between two UAVs are X and Y, respectively, and the transmission range is T, then two neighboring UAVs at different levels can communicate with each other if $X<\sqrt{T^{2}-Y^{2}}$.

X can be computed by following the Poisson point process for the UAVs of different sub-UAANETs while using an exponential distribution having a parameter equal to 2σ. Figure 2b shows the superposition of two sub-UAANETs using the Poisson point process. Therefore, the probability of a crosslink existing between two UAVs of neighboring sub-UAANETs can be estimated as:

(5) $$P=\frac{1}{2}P(X<\sqrt{T^{2}-Y^{2}})\;=\frac{1}{2}\int_{0}^{\sqrt{T^{2}-Y^{2}})}2\sigma e^{2\sigma X}dX\;=\frac{1}{2}\left(1-e^{2\sigma \sqrt{T^{2}-Y^{2}})}\right)$$

Then, the total number of crosslinks n can be computed as:

(6) $$n=\overset{N-1}{\underset{i=1}{\sum }}Z_{i}$$

where N is the UAV density and $Z_{i}$ is an indicator variable equal to 1 if there is a crosslink between two UAVs; otherwise $Z_{i}$ is equal to 0. According to the Poisson process, the mean and variance of n can be computed as:

(7) $$E\left(n\right)=Var\left(n\right)=\sigma X\left(1-1-e^{2\sigma \sqrt{T^{2}-Y^{2}})}\right)$$

According to the Chebyshev inequality,

(8) $$P\left(n=0\right)\leq P\left\{\left|n-E\left(n\right)\right|\geq E\left(n\right)-1\right\}\leq \frac{Var\left(n\right)}{{\left(E\left(n\right)-1\right)}^{2}}$$

By substituting Equation (7) into Equation (8), the probability of connectivity if neighboring sub-UAANETs have at least one cross link can be expressed as:

(9) $$P\left(n\geq 1\right)\geq 1-\frac{\sigma R\left(1-e^{-2\sigma \sqrt{T^{2}-Y^{2}}}\right)}{{\left(\sigma R\left(1-e^{-2\sigma \sqrt{T^{2}-Y^{2}}}\right)-1\right)}^{2}}$$

Let

(10) $$T^{2}=\left(1+\delta \right)Y$$

where δ is a small positive parameter. Using Equation (9) we can compute

(11) $$\underset{T\to \infty }{\lim}P\left(n\geq 1\right)\geq 1$$

Therefore, for sufficient range $T^{2}$, the probability of connectivity is almost 1 when there exists at least one crosslink between two neighboring sub-UAANETs. By combining $T^{1}$ and $T^{2}$, the connectivity of a route i can be computed as:

(12) $$Co^{i}=\max\left\{\left(1+\delta \right)Y,\;\frac{\left(1+\rho \right)\ln\left(\sigma R\right)}{\sigma }\right\}$$

where δ and ρ are two small positive parameters. It is clarified that these are parameters for controlling probability density functions considered in the mathematical modeling of the proposal in terms of maximum and minimum values or, in other words, as range constraints for these functions.

### 3.3. Service-Oriented Route Lifetime

The route lifetime between two non-neighboring dynamic mobility-centric nodes can be described as the minimum link lifetime among intermediate nodes on the route [39,40]. Assume node j and k are two intermediate nodes on the route and also lie in the transmission range T of each other. Let $\left(x^{j},\;y^{j}\right)$ and $\left(x^{k},\;y^{k}\right)$ be the coordinates of node j and k, respectively. Let $v^{j}$ and $v^{k}$ be speeds, and $\theta^{j}$ and $\theta^{k}$ the moving angles of node j and k, respectively, where $0\leq \theta^{j},\;\theta^{k}\leq 2\pi$. If a link lifetime between node j and k is $l_{jk}^{t}$, then $l_{ij}^{t}$ is formulated as:

(13) $$l_{jk}^{t}=\frac{-\left(pq+rs\right)\pm \sqrt{\left(p^{2}+r^{2}\right)a^{2}-{\left(ps-qr\right)}^{2}}}{p^{2}+r^{2}}$$

where $p=v^{j}\cos\theta^{j}-v^{k}\cos\theta^{k}$, $r=v^{j}\sin\theta^{j}-v^{k}\sin\theta^{k}$, $q=x^{j}-x^{k}$, and $s=y^{j}-y^{k}$. Here, $l_{jk}^{t}$ becomes ∞ when $\theta^{j}=\theta^{k}$ and $v^{j}=v^{k}$. Then lifetime of route i $\left(L^{i}\right)$ can be expressed as:

(14) $$L^{i}=\min\left\{l_{jk}^{t}\right\}$$

**Figure 3:**
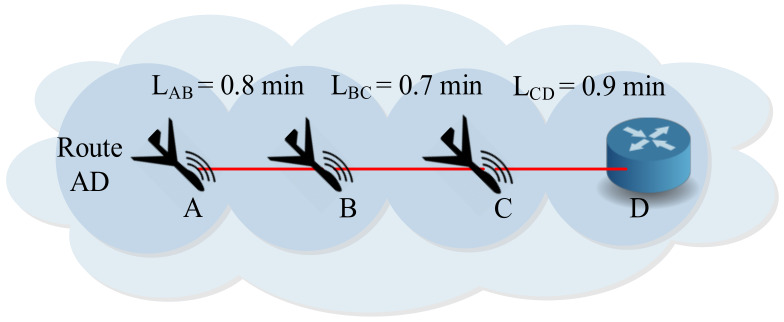
Service-oriented UAV route lifetime calculation.

According to Figure 3, the minimum link lifetime in this route is considered the route lifetime of the route AD. That is to say:

$$L^{AD}=L^{BC}=0.7\;\min$$

### 3.4. Service-Oriented UAV Route Delay

**Figure 4:**
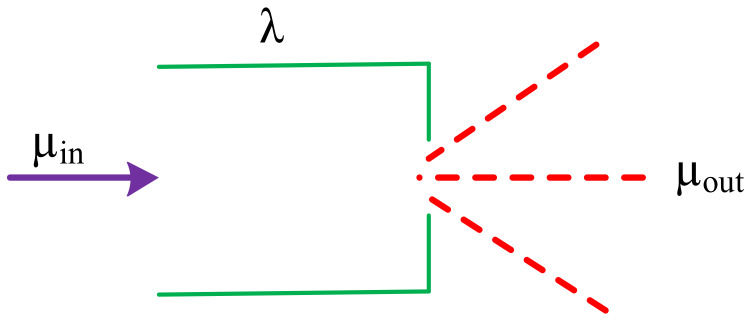
Leaky bucket strategy for UAV-centric communication.

UAV route delay is defined as the time required to send a data packet from the source UAV node to the destination UAV node. It is clarified that the UAANET is an attempt to realize an ad hoc networking scenario among the group of flying UAVs. The research theme has a large number of smart application benefits in the case of an established product for managing a UAANET environment. The networking configuration is based on a range of location-centric mobility models where each UAV can fly to support networking for a specific smart service. It is a challenging to realize such a networking scenario. However, the research community can make it possible via similar progressive research efforts. Usually the main factors in route delay are queuing delay, propagation delay, and transmission delay, which are based on scheduling techniques, traffic control schemes of a UAV node’s residual link bandwidth, processing power of ports, and traffic characteristics. Here, a leaky bucket control strategy, as illustrated in Figure 4, is utilized to control the communication volume of nodes. Assume λ is the bucket capacity, $\mu_{in}$ is the input flow rate, and $\mu_{out}$ is the service rate. As the service rate at each UAV node varies, so the highest data flow of the link is dependent on the UAV node which has the lowest service rate; therefore, $\mu_{out}=\min\left\{\mu_{out}^{1},\mu_{out}^{2},\;\mu_{out}^{3},\;\ldots \ldots ,\;\mu_{out}^{n}\right\}$. If d is the queuing delay then, according to the leaky bucket strategy, the following can be derived:

(15) $$\lambda +\mu_{in}d<\mu_{out}d$$

hence:

(16) $$d=\frac{\lambda }{\mu_{out}-\mu_{in}}$$

For the links, $\lambda =\rho -nS^{max}$, where ρ is the sudden traffic depending on the network and $S^{max}$ represents the maximum packet size, then the queuing delay is expressed as:

(17) $$d=\frac{\rho -nS^{max}}{\mu_{out}-\mu_{in}}$$

Let x be the link length between two nodes $V_{j}$ and $V_{k}$, and c be the propagation speed. Then, the propagation delay $p_{j}$ of link *j* is expressed as:

(18) $$p_{j}=\frac{x}{c}$$

The probability density function of $p_{j}$ is computed as:

(19) $$f\left(p_{j}\right)=a\times \frac{x}{c}$$

where a is constant

$$\int_{µ}^{Y}a\times \frac{x}{c}dx=1$$

$$x=\frac{2c}{Y^{2}-{µ}^{2}}$$

Now, $f\left(p_{j}\right)$ can be expressed as:

(20) $$f\left(p_{j}\right)=\frac{2x}{Y^{2}-{µ}^{2}}$$

Then, the expected propagation delay $E\left(p_{j}\right)$ can be expressed as:

(21) $$E\left(p_{j}\right)=\int_{µ}^{Y}\frac{x}{c}\times f\left(p_{j}\right)dx\;=\frac{2\left(Y^{2}+Yµ+{µ}^{2}\right)}{3c\left(Y+µ\right)}$$

If $D^{i}$ is the total delay of route i, and $B_{j}$ is bandwidth of link *j*, then:

(22) $$D^{i}=\frac{\rho -E_{hc}\times S^{max}}{\mu_{out}-\mu_{in}}+\overset{E_{hc}}{\underset{j=1}{\sum }}\frac{S^{max}}{B_{j}}+\overset{E_{hc}}{\underset{j=1}{\sum }}E\left(p_{j}\right)$$

### 3.5. Route Cost Function

In order to find the optimal route, there should be some route constraints for an UAV in the UAANET. These route constraints are as follows. In the QSPU protocol, all of the QoS metrics route connectivity, route lifetime, and route delay are merged for the purpose of finding the feasible route. The route cost function of a feasible route for an UAV can be formulated as:

(23) $$C^{r}=\alpha \left(\frac{1}{Co^{i}}\right)+\beta \left(\frac{1}{L^{i}}\right)+\gamma \left(D^{i}\right)$$

where α, β, and γ are weight factors corresponding to all three QoS metrics, subject to α+β+γ=1, and {0≤α,β,γ≤1}.

### 3.6. Service-Oriented Aerial Routing in UAANETs

**Figure 5:**
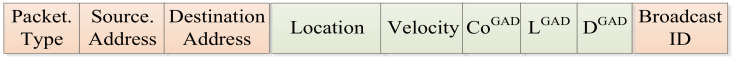
Structure of Internet Gateway (IG) advertisements (IGAD) message.

In this section, the best advertisement-based forwarding (BADF) technique is discussed, with the involvement of three aspects for controlling the overhead related to advertisement flooding. Firstly, the IP address of the originator, and the broadcast IDs of previously and newly received IG advertisement packets (IGADs) are checked by an UAV. If the UAV finds duplicate IGADs with a similar originator IP address and broadcast ID of previously and newly received IGADs, then the duplicate IGADs are discarded by this UAV. Hence, the congestion caused by duplicate IGADs is avoided based on the advertisement flooding in the network. Secondly, the UAVs that have not yet taken off or have already landed (having zero velocity) discard all the received IG advertisements. These UAVs are not involved during routing table computation. Therefore, this results in a form of limited broadcasting and reduction in network congestion. Finally, a UAV rebroadcasts the IGADs with route connectivity and route lifetime higher than a threshold value and route delay lower than a threshold value. Hop count between UAV node and IG should be lower than the maximum hop count. Hence, this minimizes the traffic overhead caused by broadcasting advertisement. In this protocol, IGs broadcast IG advertisement packets (IGADs) for advertising their QoS metrics $\left(Co,L,D\right)$ periodically inside the IoT network. Further, the UAV node knows the information of the parameters on the basis of IGADs. In this protocol, we assume all IGs have the same IGAD interval. Let IGADs for Co,L and D be $Co^{GAD}$, $L^{GAD}$ and $D^{GAD}$. $T^{s}$ is the timestamp or time at which the packet is sent. The format for the IGAD message is illustrated in Figure 5.


**Algorithm 1** Service-oriented aerial routing in UAANETs**Input:** Loc, V, $Co^{GAD}=0$, $L^{GAD}$, $D^{GAD}=0$, $B^{id}$;
**Process:**
IGAD (Loc, V, $Co^{GAD}=0$, $C^{GAD}$, $D^{GAD}=0$, $B^{id})$
  IG sends IGADs periodically.
  UAV node j receives IGAD packet.
  **if** received packet based on BADF scheme condition then
   Node j computes $Co^{j}$, $L^{j}$ and $D^{j}$ according to Equations (12), (13) and (22).
   **if** $(Co^{GAD}=0$ || $Co^{j}<Co^{GAD})$ then
    
$Co^{GAD}=Co^{j}$
   **end if**
   **if** $\left(L^{j}=0||L^{j}<L^{GAD}\right)$ then
    
$L^{GAD}=L^{k}$.
   **end if**
   
$D^{GAD}=D^{GAD}+D^{j}$.
   Update IGAD packet while replacing, Loc and V with $Loc_{j}$ and $V_{j}$, and updating $T^{s}$.
   Update route QoS metrics $\left(Co,L,D\right)$ in routing table of node j.
   Forward IGAD packet based on BADF scheme
**else** discard IGAD packet;
**end if**
Compute $C^{r}$ for each route r according to Equation (23) from routing table of UAV node

$C=\min\left\{C^{r}\right\}$
Select the route with C
Select the IG with C
**Output:** Optimized route and IG




**Figure 6:**
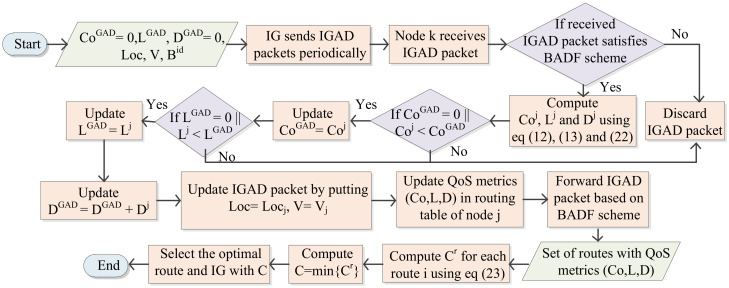
Optimal route and Internet gateway (IG) selection process of QSPU.

After receiving an IGAD packet, the UAV node estimates the value of $Co^{j}$, $L^{j}$, and $D^{j}$ on the basis of Equations (18), (20) and (23). If the values of $Co^{j}$ and/or $L^{j}$ are lower than the values of $L^{GAD}$ and/or $C^{GAD}$, then the QoS metrics $\left(Co,L,D\right)$ are updated in the UAV’s routing table and the parameters X, Y, V, and $T^{s}$ are updated in the IGAD packet; otherwise, the current $L^{GAD}$ and/or $C^{GAD}$ are utilized in the routing table and IGAD. Further, the value of $D^{GAD}$ is updated by adding the value of $D^{j}$. Then, on the basis of the BADF technique, the IGAD packet is further forwarded inside the network. The basic procedure for route selection is presented by Algorithm 1. The message for route updating is transmitted to the source UAV node by the intermediate UAV node, if there is a possibility of novel link establishment or current link breakage along the UAV’s route. In this way, based on updated route parameters, the source UAV node decides on a potential route to transmit the packet. The UAV node preserves the records of the QoS parameters for each route to IGs in its routing table. The optimal route and IG selection process of the QSPU protocol is also presented in Figure 6.

## 4. Experimental Results and Discussion

In this section, simulation experiments are performed to carried out performance analysis of the of the proposed quality of service provisioning framework for UAVs (QSPU) in a software-based UAANET environment. Simulation settings, parameters, and comparative analysis of results are discussed for the UAANET-assisted IoT environment. The implementation of the proposal and some state-of-the-art techniques was conducted using the network simulator (ns-2) environment [41]. The major simulation settings include 200 UAV wireless nodes enabled by the 802.11b version of Wi-Fi in a 3D simulation space of 1000×1000×1000 m. A wireless transmission range of 200 m was assumed for UAV-to-UAV and UAV-to-ground communication, with most service-oriented computing performed at the ground station server and UAVs active in the UAANET environment. The communication link bandwidth for UAV-to-UAV communication was assumed to be 5 Mbps and UAV-to-ground server was 10 Mbps. A total time of 45 min was simulated for each experiment performed in the simulator. Each data point considered in the experimental results represents an average of 10 simulation experiments performed under similar parameter settings to avoid objectivity and provide normalization in results analysis.

Understandably, for the sake of greater clarity and reproducibility of experiments, previous researchers have highlighted the significance of the type of UAVs considered in the experiment, in addition to collision handling mechanisms. It is noted that wireless communication-enabled nodes were considered as UAVs that can take location coordinates from a range of values. This range of values was considered different for all the UAV nodes for managing the risk of collision in the simulation network area, given that a large number of UAVs in a small network area could result in collisions among UAVs. However, by maintaining a location server, which allocates different a range of coordinates for each UAV, the possible collision issue can be handled effectively with reduced UAV mobility constraints. Further, it is also clarified that, in the simulation, 802.11b Wi-Fi was utilized for its suitability and availability in the ns2 network simulation environment. The standard supports network speed of approximately 11 Mbps, which was sufficient for the considered network settings in our implementation. For comparative performance analysis, several traditional ad hoc networking frameworks and a recent UAV framework were considered, including AODV, GPSR, and HCU. Further, the range of metrics considered in the performance analysis included smart service delivery ratio, UAV connectivity, number of handoffs among UAV ad hoc networks, service overhead, and service delay. The mathematical modeling derived in Section 3 forms the basis of these metrics in the analysis of results. Similar metrics were also considered in a recent development in the literature on UAV environments [36]. A summarized list of major simulation configuration settings is shown in Table 2.

### 4.1. Analysis of Results with Similar Weighting Factor

**Figure 7:**
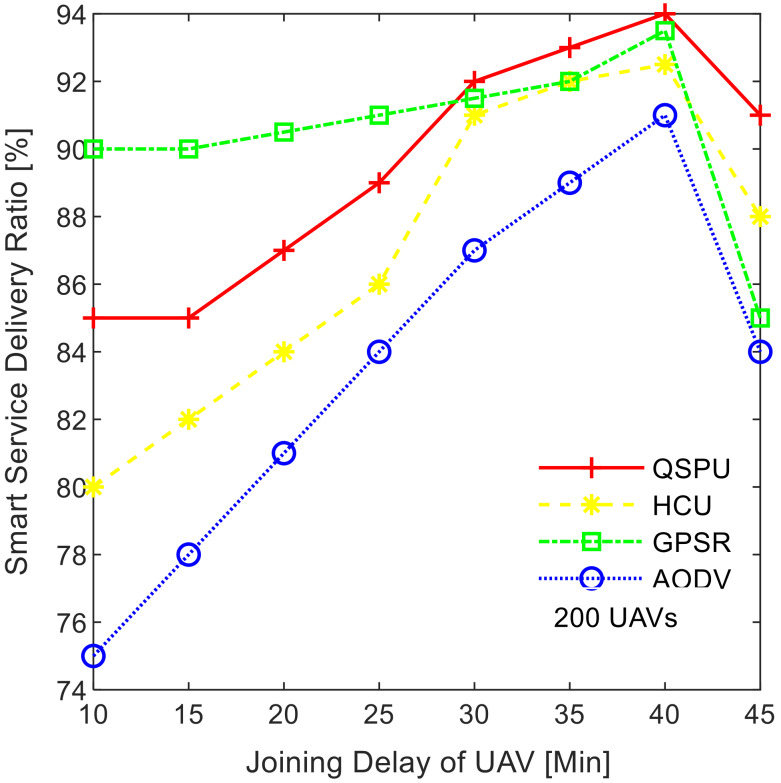
Impact of joining delay of UAVs on smart service delivery ratio (SSDR).

Two scenarios, including experimental results with the same weight factors and experimental results with varying weight factors, are considered to compare the performance of the proposed QSPU framework with that of the state-of-the-art protocols. In this section, experimental results are described while assigning equal weight factors for all the metrics α=β=γ=0.33. The smart service delivery ratio (SSDR) is described as the ratio of the successfully transmitted packets to the total number of transmitted packets. Figure 7 shows the variation in SSDR as the joining delay of UAVs increases for all of the compared protocols, i.e., AODV, GPSR, HCU, and QSPU. According to the results in Figure 7, from the outset until the joining delay threshold value of 40 min, the SSDR gradually increases for all of the compared protocols. However, when the joining delay increases beyond 40 min, then the SSDR falls for each protocol. QSPU performs better than the state-of-the-art protocols, AODV, GPSR, and HCU, for joining delays that are greater than 30 min. In the case of AODV and HCU, overhead increases rapidly, therefore SSDR falls quickly after the threshold. For QSPU, unstable routes are discarded precisely, therefore SSDR is higher than that of the state-of-the-art techniques.

**Figure 8:**
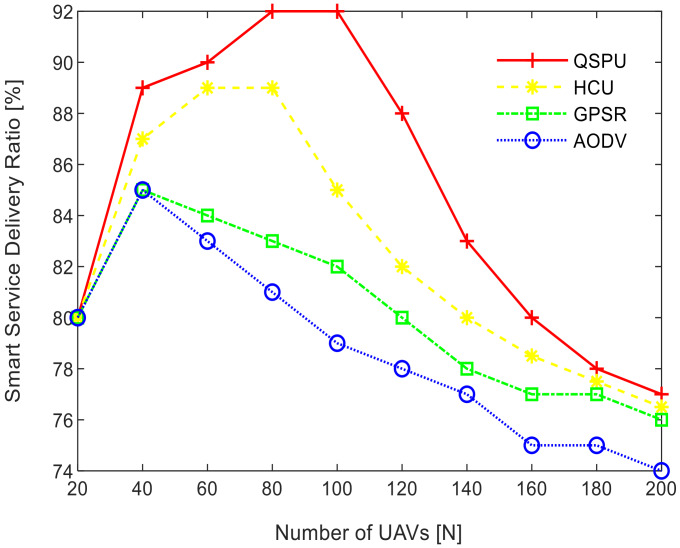
Impact of number of UAVs on smart service delivery ratio.

**Figure 9:**
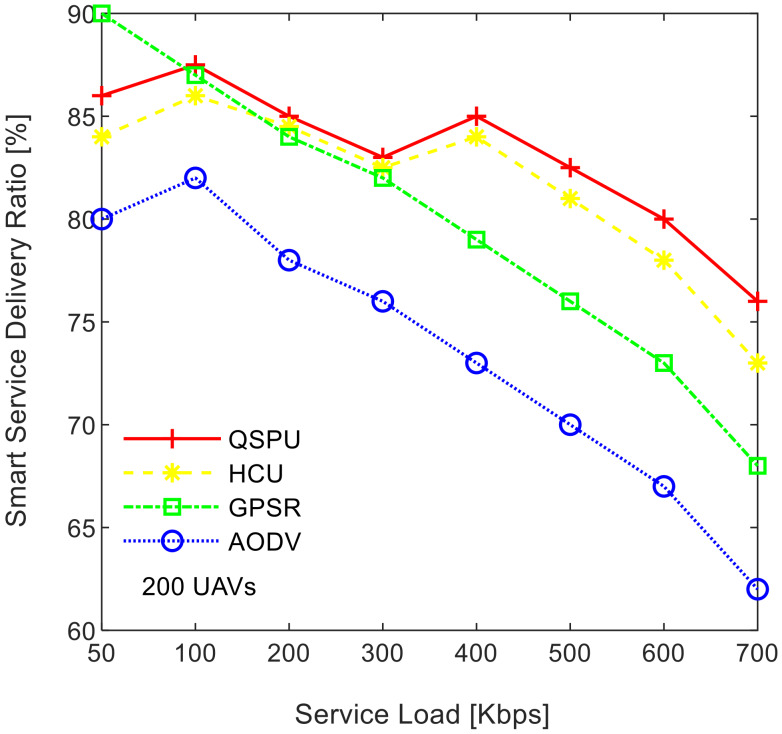
Impact of service load on smart service delivery ratio.

The results in Figure 8 show the variation in smart service delivery ratio with the increment in number of UAVs in the network for all of the considered protocols. In the case of AODV, GPSR, and HCU, the delivery ratio fell gradually when the number of UAVs reaches 40. However, for QSPU, it begins to fall when the number of UAVs reaches 100, and with a slower rate of decline. From the results, it is clearly illustrated that the service delivery ratio for QSPU is far higher as compared to that of AODV, GPSR, and HCU, because QSPU can select the next hop with the shortest queue of packets among all of the possible UAVs nodes. Thus, this results in the avoidance of local blocking of services at UAVs. Further, the impact of the number of UAVs on the smart service delivery ratio is noteworthy. Initially, with an increasing number of UAVs, the smart service delivery ratio increases. However, the service delivery ratio begins to deteriorate as the number of UAVs added to the network increases from 100 towards 200. This can be attributed to the occurrence of network partitioning, and the possibility of a sender that is connected to the ground service and receiver UAVs that are in different aerial ad hoc networks. The growing number of UAVs results in different aerial ad hoc networks. This reduces the smart service delivery ratio to approximately 75%, which can be further verified from the results in Figure 9 where the service delivery ratio falls below 70% with higher service load.

**Figure 10:**
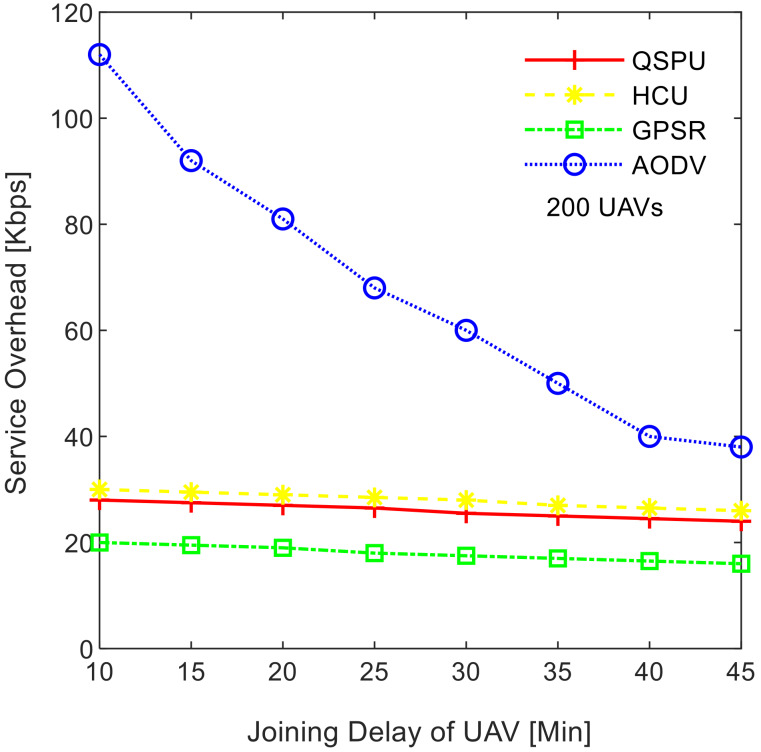
Impact of joining delay of UAVs on service overhead of flying ad hoc networks.

Figure 9 illustrates the impact of service load variation on the smart service delivery ratio, while maintaining the number of UAVs at 200. As shown in Figure 9, the service delivery ratio decreases as traffic load increases for all of the compared protocols. The ratio in the case of the proposed QSPU is higher than that of AODV, GPSR, and HCU because route load balancing is considered in the proposed QSPU but not in the state-of-the-art protocols. In the case of QSPU, the delivery ratio is 6.5% and 11.5% higher than the delivery ratio in the case of GPSR, HCU, and AODV, when traffic load is 600 Kbps. However, when the traffic load is 700 Kbps, the delivery ratio for QSPU is 8% and 13% higher than the delivery ratio for GPSR, HCU, and AODV. The overhead is considered the number of excess packets generated for the successful transmission of the actual number of packets from the source to the destination in a specific smart service. The results in Figure 10 illustrate the variation in the overhead with joining delay time increment. Due to utilization of best advertisement-based forwarding to minimize overhead in the case of QSPU, the overhead is lower than that of AODV and HCU. However, GPSR results in less overhead than in QSPU, AODV, and HCU because GPSR utilizes smaller periodic HELLO packets for neighbor discovery compared to the IGAD packets of QSPU, which do not guarantee quality of service in a flying ad hoc network environment.

**Figure 11:**
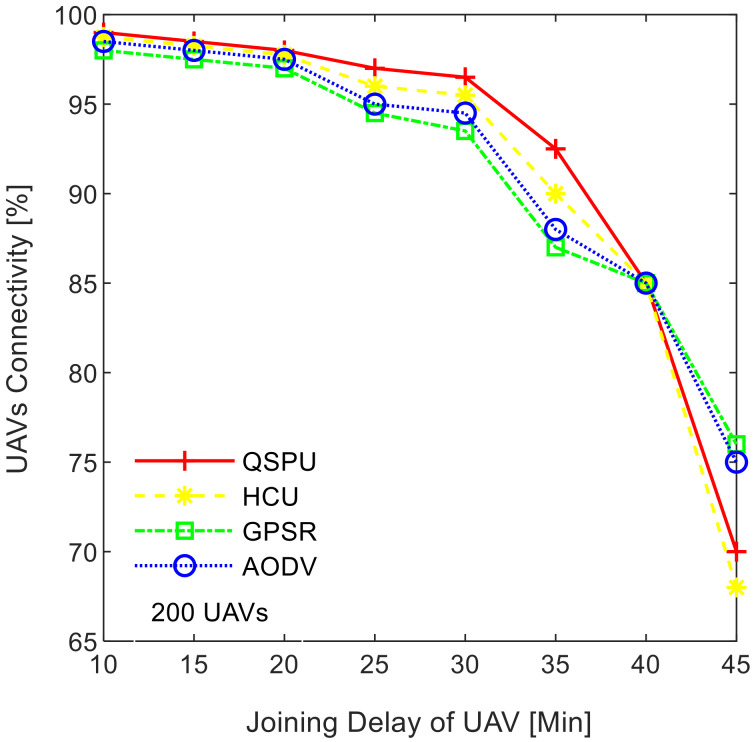
Impact of joining delay of UAVs on the connectivity in UAANETs.

**Figure 12:**
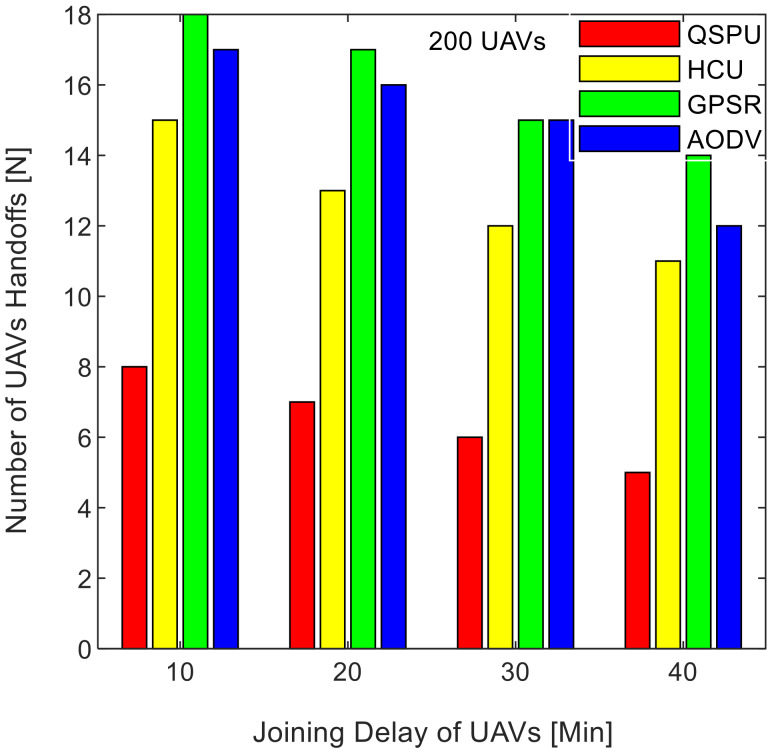
Impact of joining delay of UAVs on number of handoffs in UAANETs.

**Figure 13:**
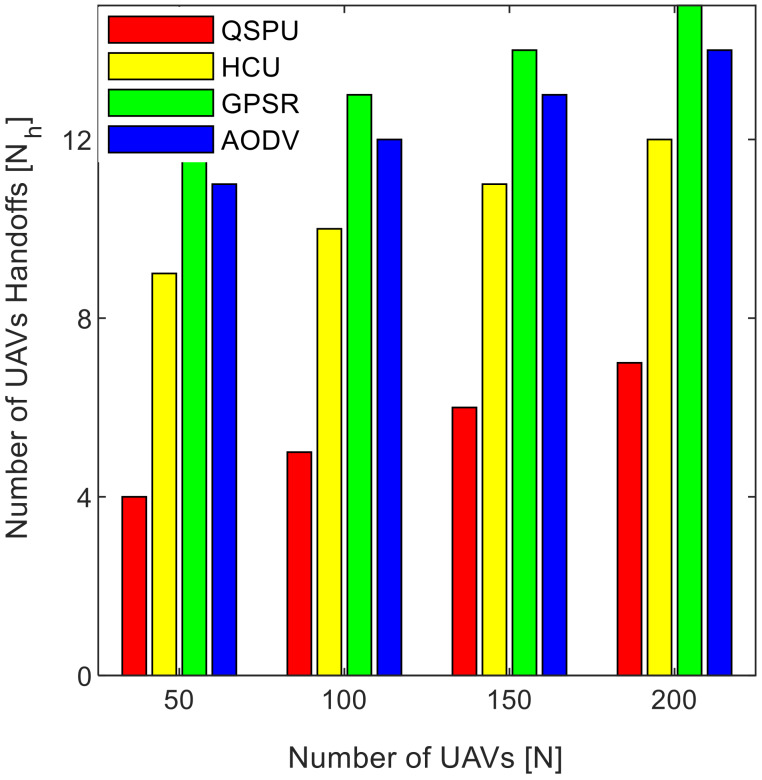
Impact of number of UAVs on number of handoffs in UAANETs.

**Figure 14:**
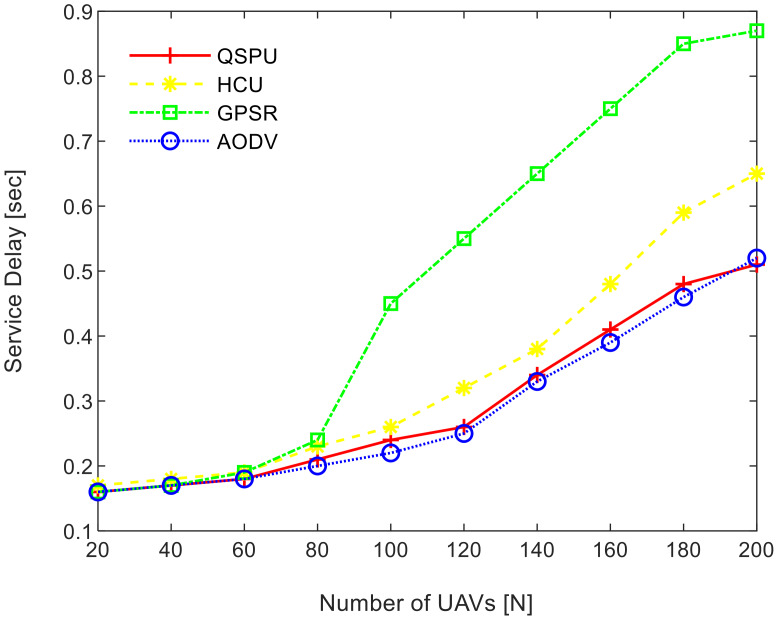
Impact of number of UAVs on service delay in UAANETs.

Connectivity among UAVs is considered the fraction of directly connected UAV nodes having at least one UAV with multiple routes to a ground station service provider. The results in Figure 11 show the variation in connectivity with the variation in joining delay of UAVs. It can be observed that as the joining delay of UAVs increases, the connectivity decreases for all the compared protocols. The connectivity reaches almost 100% for compared protocols, when joining delay is equal to or less than 20 min. When joining delay is lower than 40 min, QSPU outperforms the state-of-the-art protocols, whereas, for more than 40 min, the performance of QSPU weakens, because of strict IGAD forwarding conditions to build up the routes. Stability is inversely proportional to the number of handoffs. Figure 12 illustrates the impact of joining delay on the average number of handoffs for all three considered protocols. QSPU performs better than the state-of-the-art protocols, with a lower number of handoffs, because QSPU utilizes better path duration for new route selection, whereas no path stability metric is considered in the case of AODV, HCU and GPSR. The results in Figure 13 show the variation in stability in terms of the average number of handoffs when the number of UAVs is 50, 100, 150, and 200. It is clearly shown that stability decreases as the number of UAVs increases in the case of all the compared protocols. Further, with the increment in the number of UAVs, handoffs are slower and the increment in the number of handoffs is lower in the case of QSPU compared to AODV and GPSR. This is because the greater the number of UAVs, the larger the air communication traffic, and, as a result, QSPU is more likely to find the next hop with more stability and with less node delay. In contrast, AODV utilizes hop count as the only metric, and AODV and GPSR both always find the shortest path, which does not ensure link stability. Consequently, UAVs reach their maximum range more frequently, which is prone to cause handoff. Route delay is considered as the time required to transmit a data packet from source node to destination node. Figure 14 shows the impact on route delay with the variation in the number of UAVs. Route delay in the case of QSPU is lower than that of GPSR because route delay is considered one of the route selection metrics in QSPU, but not in GPSR. QSPU also considers local dynamic queue delay for nodes, which avoids congestion, whereas QSPU has a slightly higher route delay compared to AODV because, although packets are forwarded through the shortest path in the case of AODV, it is unstable.

### 4.2. Analysis of Results (With Varying Weight Factors)

**Figure 15:**
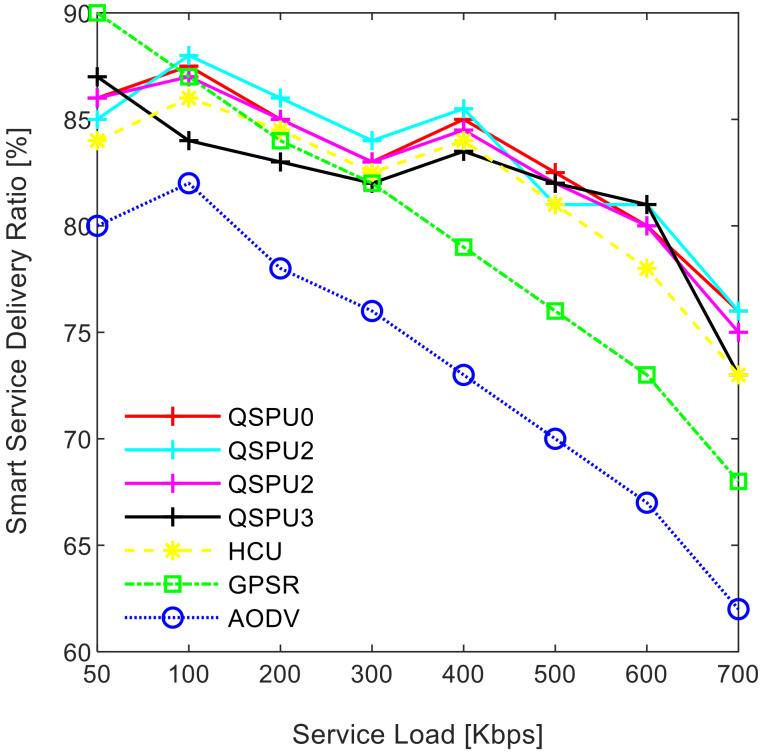
Variation in SSDR with service load.

The performance of the proposed QSPU was optimized while performing the simulation with varying weight factors corresponding to route metrics. Three scenarios are presented in the simulation for this purpose. In the first scenario, the highest priority is given to the route connectivity metric while assigning weight factors as: α=0.7, β=0.15, and γ=0.15. In the case of this scenario, the protocol is labelled QSPU1. In the next scenario, the metrics are as follows: route lifetime is prioritized, and weight factors are considered as α=0.15, β=0.7, and γ=0.15; this scenario protocol is labelled QSPU2. In the third scenario, the metrics are: route delay is preferred, and weight factors are α=0.15, β=0.15, and γ=0.7; the protocol in the third scenario is labelled QSPU3. Further, the performance of QSPU1, QSPU2, and QSPU3 are compared with QSPU0 (without weights), AODV, HCU, and GPSR protocols. A comparison of SSDR between QSPU in all scenarios, and the state-of-the-art protocols with varying traffic load is presented in Figure 15. The performance of QSPU0, QSPU1, QSPU2, and QSPU3 is almost the same in terms of SSDR, while these scenarios perform better that the state-of-the-art protocols of GPSR, HCU, and AODV. It is clearly illustrated that stability and load balancing must be prioritized for heavy traffic in order to improve SSDR.

**Figure 16:**
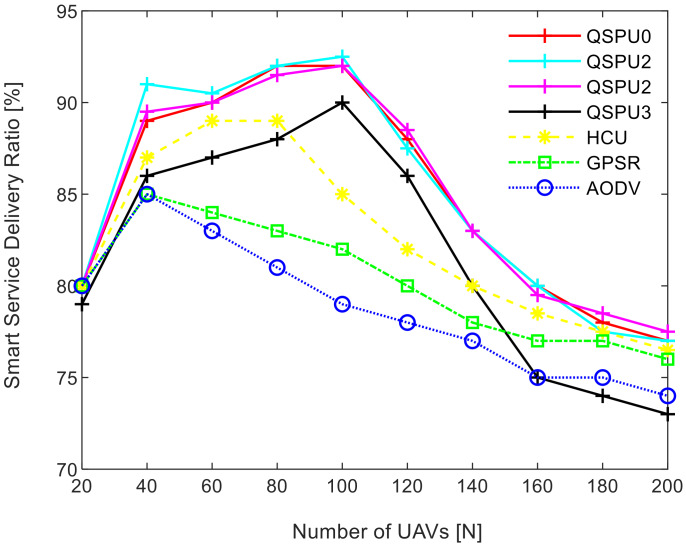
SSDR vs. number of UAVs.

**Figure 17:**
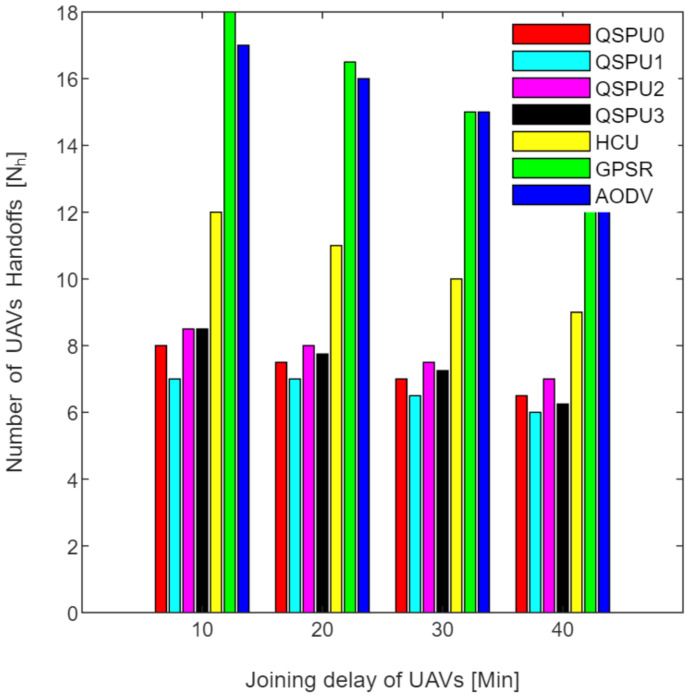
UAV handoffs vs. joining delay of UAVs.

The results in Figure 16 illustrate the variation in SSDR with the increment in the number of UAVs for all scenarios of QSPU. The SSDR in the case of QSPU0, QSPU1, and QSPU2 is almost equal but lower in the case of QSPU3. However, the performance of QSPU in all scenarios is better than that of AODV, HCU, and GPSR because QSPU can select the next hop with the shortest queue of packets among all of the possible nodes. Thus, this results in the avoidance of local blocking. Figure 17 shows the behavior of QSPU in all scenarios in terms of the number of handoffs (stability) while varying the joining delay of UAVs. In the case of QSPU1, the number of handoffs is slightly lower compared to that of QSPU0, but the number of handoffs is slightly higher in the case of QSPU2 and QSPU3 than that of QSPU0. However, QSPU in all scenarios performs considerably better than GPSR and AODV in terms of handoff count.

**Figure 18:**
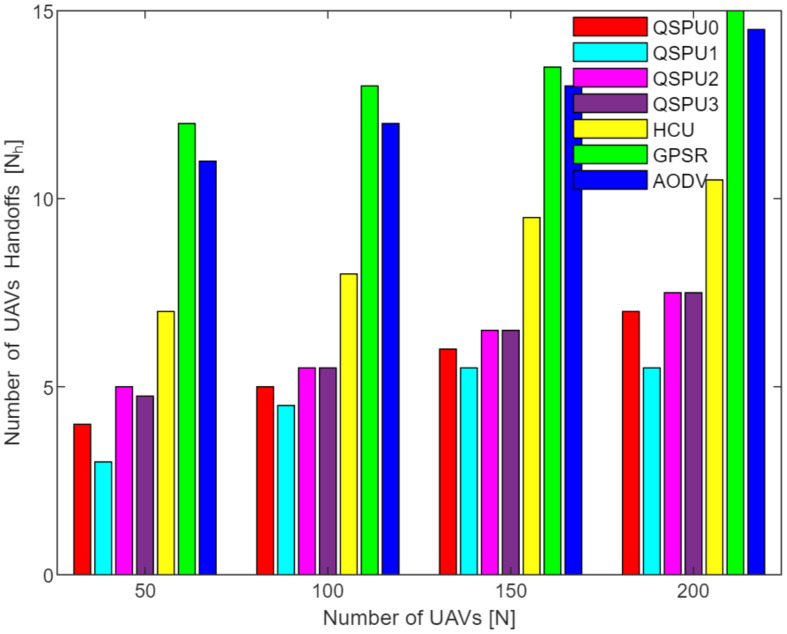
UAV handoffs vs. number of UAVs.

**Figure 19:**
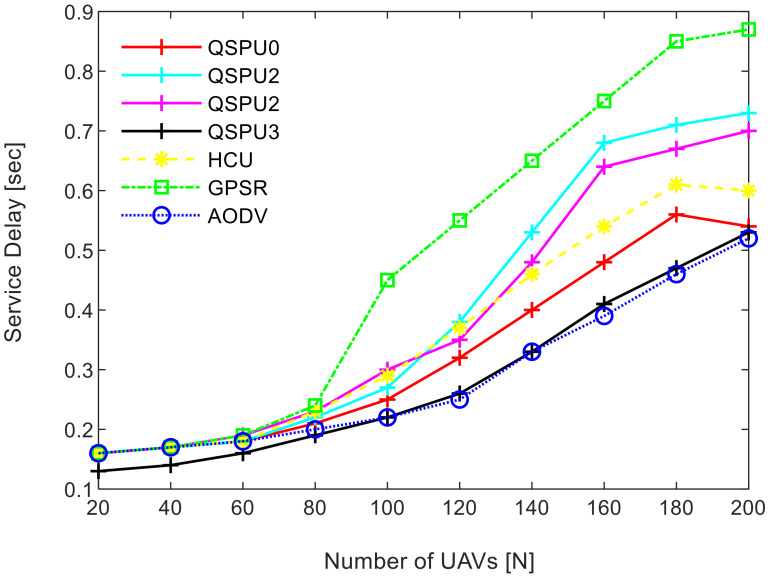
Service delay vs. number of UAVs.

Figure 18 shows the stability as measured by handoff count when the number of UAVs is simulated as 50, 100, 150, and 200 in all scenarios of QSPU. It is clearly shown that the number of handoffs for QSPU1 is the least, while QSPU0, QSPU2, and QSPU3 results are similar but slightly higher. Nonetheless, the QSPU in all scenarios performs significantly better than the state-of-the-art protocols, AODV, HCU, and GPSR, in terms of number of handoffs. The results in Figure 19 illustrate the variation in route delay with the increment in the number of UAVs for all the scenarios of QSPU. From Figure 19 it is clearly shown that QSPU3 outperforms QSPU0, QSPU1, QSPU2, and GPSR, because in QSPU3 highest priority is given to route delay, with a corresponding weight factor equal to 0.7. However, QSPU3 has a slightly higher route delay compared to AODV because, although packets are forwarded through the shortest path in the case of AODV, it is unstable. The results considering various weight factors show the enhancement in route stability, route delay, and packet delivery ratio considering various scenarios inside the network.

## 5. Conclusions and Future Work

In this paper, a quality of service provisioning protocol for a UAANET (QSPU) environment is presented. The aim is the enhancement of network performance in a UAV-assisted IoT environment. A mobility model is presented for deriving the probability of connectivity between two sensor-enabled UAV nodes inside a sub-UAANET and between two sub-UAANETs. Further, a route selection algorithm based on three service parameters, namely, UAV connectivity, service-oriented route lifetime, and service-oriented UAV route delay is developed by utilizing the BADF technique to minimize the overhead related to traffic. Through simulation experiments, the performance of the proposed QSPU with respect to the state-of-the-art protocols, GPSR and AODV, is analyzed and compared in terms of smart service delivery ratio, UAV connectivity, UAV stability, service overhead, and service delay. The simulation results show that QSPU outperforms the state-of-the-art protocols.

In future research, the authors will investigate the potential of the proposed UAV-assisted framework in on-road vehicular traffic environments with the aim of enabling smart traffic services [42]. Further, the introduction of energy utilization as a new performance criterion, with the aim of reducing the cost of operations and human interventions in sensor-enabled UAV-assisted services, would be a framework design challenge [43]. Finally, system testing for an underwater UAV environment could be a further topic for future research [44].

## Figures and Tables

**Table 1:** Notations.

Notation	Description	Notation	Description
T	Transmission range	$l_{ij}^{t}$	Link lifetime
$P^{c}$	Connectivity probability	$L^{i}$	Lifetime of route i
X	Horizontal distance	$C^{r}$	Route cost function
$\theta^{j}$	Moving direction of node j	$D^{i}$	Total delay of route i
Y	Vertical distance	*Co*	Route connectivity
n	Number of cross links	L	Route lifetime
$x^{j}$	X coordinate of node j	D	Delay
$y^{j}$	Y coordinate of node j	$T^{s}$	Time stamp
$v^{j}$	Velocity of node j	$B^{id}$	Broadcast ID

**Table 2:** Simulation setup.

Parameters	Values	Parameters	Values
3D Simulation space	1000×1000×1000 m	Medium Access Control (MAC) protocol	Time Division Multiple Access (TDMA)
Simulation time	45 min	Constant Bit Rate (CBR) packet size	512 bytes
Trans/Receive antenna	Omnidirectional	CBR interval	0.01 s
IGAD interval	Uniform (3.5,4.5) s	UAV-UAV link bandwidth	5 Mbps
UAV-UAV range	200 m	UAV-Ground link bandwidth	10 Mbps
UAV-Ground range	200 m	Packet Type	User Datagram Protocol (UDP)
Number of UAVs	200	Channel Type	Wireless
Propagation model	Free space	Wi-Fi version	802.11b
